# Tissue engineering applications of recombinant human collagen: a review of recent progress

**DOI:** 10.3389/fbioe.2024.1358246

**Published:** 2024-02-14

**Authors:** Lili Cao, Zhongfeng Zhang, Dan Yuan, Meiping Yu, Jie Min

**Affiliations:** ^1^ Department of Plastic Surgery, Zhejiang Rongjun Hospital, Jiaxing, Zhejiang, China; ^2^ General Surgery Department, Jiaxing No.1 Hospital, Jiaxing, Zhejiang, China

**Keywords:** tissue engineering, regenerative, biomaterials, recombinant human collagen, biomedicine

## Abstract

With the rapid development of synthetic biology, recombinant human collagen has emerged as a cutting-edge biological material globally. Its innovative applications in the fields of material science and medicine have opened new horizons in biomedical research. Recombinant human collagen stands out as a highly promising biomaterial, playing a pivotal role in crucial areas such as wound healing, stroma regeneration, and orthopedics. However, realizing its full potential by efficiently delivering it for optimal therapeutic outcomes remains a formidable challenge. This review provides a comprehensive overview of the applications of recombinant human collagen in biomedical systems, focusing on resolving this crucial issue. Additionally, it encompasses the exploration of 3D printing technologies incorporating recombinant collagen to address some urgent clinical challenges in regenerative repair in the future. The primary aim of this review also is to spotlight the advancements in the realm of biomaterials utilizing recombinant collagen, with the intention of fostering additional innovation and making significant contributions to the enhancement of regenerative biomaterials, therapeutic methodologies, and overall patient outcomes.

## Graphical Abstract

**Figure:**
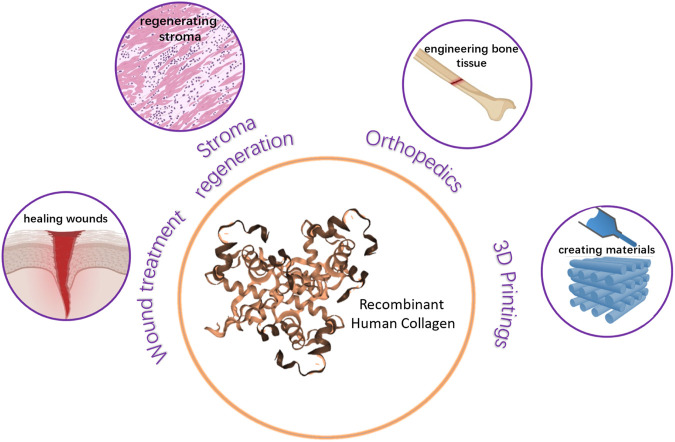
Recombinant human collagen applications in biomedical systems.

## 1 Introduction

Collagen, the most abundant protein in the extracellular matrix of animal cells, plays a pivotal role in providing structural support and regulating cellular behavior (Avila Rodríguez et al., 2018; Coppola et al., 2020). To date, 29 types of collagens have been identified, with types I, II, and III constituting over 90% of the total collagen in the human body (Meyer, 2019; Naomi et al., 2021). Collagen provides tensile strength and is the primary component of skin, bones, cartilage, and connective tissues (Law et al., 2017; Jafari et al., 2020). Due to its biocompatibility, biodegradability, and low immunogenicity, collagen has been extensively investigated and employed as a biomaterial in the field of tissue engineering and regenerative medicine (Irawan et al., 2018; Copes et al., 2019; Lin et al., 2019).

However, the majority of collagen used for biomedical applications is still derived from animal sources, such as the skin, tendons, and bones of bovines, pigs, and avian species (Avila Rodríguez et al., 2018; Felician et al., 2018). Animal-sourced collagen has inherent drawbacks. It exhibits batch-to-batch variability in quantity and quality, can potentially trigger immunogenic responses, and carries the risk of transmitting animal viruses and prions (Gauza-Włodarczyk et al., 2017a; Coppola et al., 2020). Despite the success of animal-derived collagen products like Zyderm, the pursuit of recombinant collagen aims to refine the safety and efficacy of collagen-based treatment (Shekhter et al., 2019). Produced through intricate *in vitro* techniques, recombinant collagens are designed to emulate the post-translational modifications seen in natural collagens, such as hydroxylation and glycosylation, thereby achieving a high degree of similarity to human collagen (Kim et al., 2017). Recombinant human collagen produced via biotechnological methods can overcome these limitations associated with xenogeneic or allogeneic collagen (Wang et al., 2022). Over the past few decades, research on recombinant collagen has made significant strides in genetic recombination, protein expression, and material preparation (Ma et al., 2022). This article provides a comprehensive review of the latest advancements in recombinant human collagen and its applications as biomaterials in tissue engineering and regenerative medicine.

Collagen possesses a characteristic triple-helical structure, composed of three polypeptide chains known as α-chains (Mi et al., 2018; Rappu et al., 2019). *In vitro* studies have recombinantly produced different types of collagens from isolated genes, including types I, II, III, and V collagen (Woodley et al., 2017; Doan et al., 2019; Shuai et al., 2023). Compared to natural collagen, recombinantly produced collagen achieves proper post-translational modifications, including hydroxylation and glycosylation (Shekhter et al., 2019; Deng et al., 2021). Based on the composition of α-chains, recombinant collagen can be categorized into homotrimeric (I, II, III), heterotrimeric (XI), and hybrid forms (IX) (Ferraro et al., 2017; Chen et al., 2020). Type I collagen is the most abundant type in many tissues, while type III collagen is relatively less abundant but plays a crucial role in maintaining tissue integrity and regulating scar formation (Kuivaniemi and Tromp, 2019; Di Martino et al., 2022; Harris et al., 2022). Extensive research has also been focused on type III collagen due to its therapeutic potential in promoting wound healing and tissue regeneration (Zhang Wei et al., 2018a; Xia et al., 2018; Davison-Kotler et al., 2019).

To produce recombinant collagen, expression systems including mammalian, insect, yeast, and bacterial cells have been explored (Capella-Monsonís et al., 2018; Davison-Kotler et al., 2019). Mammalian cells like CHO and HEK293 have translation mechanisms most similar to human cells and can therefore produce collagen with correct modifications (Gauza-Włodarczyk et al., 2017b; Lim et al., 2019). However, their relatively low yield and high cost hinder industrial-scale production (Felician et al., 2018; Song et al., 2018; Meng et al., 2019). Bacterial and yeast expression systems are more cost-effective but do not achieve proper post-translational processing (Amyoony et al., 2023). Therefore, strategies have been developed to enhance the quality of recombinantly produced collagen by genetically modifying host cells or supplementing post-translational enzymes. Recent research has made significant strides in optimizing expression systems and purifying large quantities of structurally native collagen (Knüppel et al., 2017; Rittié, 2017).

Following purification, recombinant collagen is fabricated into various biomaterials for biomedical applications (Zhang et al., 2019; Ghomi et al., 2021). Collagen hydrogels prepared by differential mechanisms have been extensively studied as scaffolds and drug delivery carriers (Li et al., 2020). By adjusting the hydrogel density, degree of cross-linking, and the incorporation of other biomolecules, its degradation, mechanical strength, and biological activity can be customized. The inclusion of growth factors and cells further enhances the regenerative potential of collagen hydrogels (Arakawa et al., 2017; Sarrigiannidis et al., 2021). Lyophilized collagen materials processed through freeze-drying present another format as wound dressings (Magro et al., 2017). Scaffold formats of collagen have been expanded further by various techniques including 3D printing, electrospinning, and particle sintering (Rashedi et al., 2017; Zhang et al., 2018b; Abbas et al., 2020). Functionalizing recombinant collagen biomaterials with nanoparticles, peptides, and stem cells has emerged as a promising strategy for precisely guiding tissue regeneration.

In tissue engineering applications, recombinant collagen biomaterials have been widely studied for skin regeneration due to the natural abundance of collagen in the dermis (Koons et al., 2020). Collagen hydrogels promote wound healing by stimulating cell proliferation, migration, angiogenesis, and collagen deposition (Nguyen et al., 2019; Roshanbinfar et al., 2023). When used as covers for skin grafts or wound dressings, they accelerate re-epithelialization. For load-bearing tissues, collagen scaffolds combined with stem cells hold potential in bone and cartilage regeneration. Upon implantation of collagen/stem cell constructs, substantial new bone and cartilage formation was observed in animal models (Wang et al., 2020). In vascular engineering, cell-seeded collagen tubular scaffolds have demonstrated the ability to remodel into vascular grafts (Copes et al., 2019; Minor and Kareen, 2020). New evidence has also validated the feasibility of using collagen implants to repair damaged myocardium and cornea. Looking forward, the development of optimized recombinant collagen production, functional biomaterial design, and translational research will further expand its regenerative applications (Carrabba and Madeddu, 2018; Minor and Kareen, 2020). The future of recombinant human collagen lies in overcoming the limitations of animal-sourced collagen and propelling the development in the fields of tissue engineering and regenerative medicine. Establishing stable high-yield expression systems and purification processes for industrial-scale production remains a major challenge.

This review summarizes the research on recombinant human collagen’s applications in biomedical systems, including its effects in wound treatment, stroma regeneration, and orthopedics. We explore studies on recombinant collagen-based hydrogels, scaffolds, microspheres, and dressings for healing wounds, regenerating skin, and engineering bone tissue. The review also encapsulates research on 3D printings containing recombinant collagen (Table 1). Our goal is to shed light on the advancements and inspire further innovations in recombinant collagen’s biomaterial and clinical uses, with the hope that ongoing development will improve biomaterials, therapies, and patient outcomes.

**TABLE 1:** Tissue engineering applications of recombinant human collagen.

Applications	Advantages	Challenges	Reference
Wound treatment	1. Promotes accelerated wound healing	1. Potential for immune response	Han and Roger, (2017); Koehler et al. (2018); Sun et al. (2018); Las Heras et al. (2020); Mathew-Steiner et al. (2021) Thapa et al. (2020) Muhonen et al. (2017); Kathawala et al. (2019); Su et al. (2021); Xu et al. (2022)
	2. Provides excellent biocompatibility and cell adhesion	2. Cost of production and purification can be high	
	3. Can be fabricated into various forms (e.g., dressings, hydrogels)		
Stroma regeneration	1. Supports cell proliferation and differentiation	1. Possible immunogenicity	Addi et al. (2017); Parmar et al. (2017); Quinlan et al. (2017); Sheehy et al. (2018); McPhail et al. (2020); Yang et al. (2021a); Yang et al. (2021b); Kong et al. (2022) Haagdorens et al. (2019); Wang, (2021) Jeon et al. (2017); He et al. (2018); Huang et al. (2018)
	2. Can be used to construct diverse tissue scaffolds	2. Control over mechanical properties can be challenging	
	3. Promotes skin regeneration		
Orthopedics	1. Can be used for bone tissue engineering	1. Mechanical strength may be less than some synthetic materials	Chan et al. (2017); Ramírez-Rodríguez et al. (2017); Andrews et al. (2019); Bien et al. (2020); Fushimi et al. (2020)
	2. Offers good biocompatibility and bioresorbability	2. Potential for immune response	
	3. Can potentially stimulate bone growth		
3D printing	1. Enables the creation of complex and patient-specific structures	1. Requires specialized 3D printing technology	Włodarczyk-Biegun and Del Campo, (2017); Hong et al. (2018); Lee et al. (2019); Osidak et al. (2020); Tytgat et al. (2020); Muthusamy et al. (2021) Gungor-Ozkerim et al, (2018); Isaacson et al. (2018); Gudapati et al. (2020); Cui et al. (2017); Zhang et al. (2017); Curtin et al. (2018); Nocera et al. (2018); Matai et al. (2020); Dai et al. (2021); Elalouf, (2021); Tang et al. (2021)
	2. Can be combined with other materials for enhanced properties	2. Control over mechanical properties and print resolution can be challenging	
	3. Potential for creating personalized implants		

## 2 Hydrogel delivery of recombinant collagen for chronic wounds healing

Chronic wounds, including diabetic foot ulcers, are characterized by impaired healing and persistent inflammation (Mathew-Steiner et al., 2021). The wounds become trapped in a prolonged inflammatory stage and are unable to progress through the normal phases of healing (Las Heras et al., 2020). This results in significantly delayed closure compared to acute wounds. Chronic wounds also frequently become colonized with bacteria, leading to infection. The sustained inflammatory environment causes continuous tissue breakdown and inhibits cell proliferation and angiogenesis (Han and Roger, 2017).

Recombinant collagen scaffolds offer several advantages for chronic wound treatment (Sun et al., 2018). As the major structural component of the extracellular matrix, collagen provides an ideal environment to facilitate cell migration and enable wound closure (Koehler et al., 2018; Sun et al., 2018). Recombinant collagen allows precise control over scaffold properties like porosity and bioactivity (Catanzano et al., 2021). Despite these benefits, challenges remain in optimizing delivery of recombinant collagen to improve healing. Fast degradation rates make it difficult to achieve sustained collagen presence within dynamic wound environments. Enhancing collagen scaffold stability through chemical or physical crosslinking may help prolong bioactivity but can also negatively impact integration with native tissue (Thapa et al., 2020). Therefore, effective chronic wound therapies will likely require recombinant collagen delivery platforms that balance scaffold remodeling with regeneration of functional tissue (Ahmad et al., 2021). Further research is needed to translate the promise of recombinant collagen into effective wound treatments that overcome the barriers to healing in chronic wounds.

RhCol III, the primary collagen type in early granulation tissue, shows potential for accelerating wound closure. Hydrogels composed of rhCoI lII have been developed. These hydrogels feature porous microstructure, near-physiological swelling ratios, and significant cell adhesion. *In vivo* testing in diabetic mice demonstrated expedited wound closure with rhCol III treatment compared to controls. The hydrogels provide a moist environment conducive to healing and act as an *in situ* forming scaffold for cell migration.

In a study, Wang et al. developed a specialized recombinant human type III collagen (rhCol III) and constructed a multifunctional, microenvironment-responsive hydrogel system integrating this custom rhCol III and multifunctional antimicrobial nanoparticles (PDA@Ag NPs) (Hu et al., 2021). This advanced hydrogel showcases accelerated degradation in the setting of chronic diabetic wounds, orchestrating the regulated and demand-driven release of various therapeutic agents. Initially, the hydrogel releases PDA@Ag NPs which possess potent antimicrobial activity against *Staphylococcus aureus* and *Escherichia coli*, thereby facilitating rapid bacterial eradication. Concurrently, these nanoparticles exhibit antioxidant and anti-inflammatory properties within the wound environment. Subsequently, the release of rhCol III stimulates the proliferation and migration of murine fibroblasts and endothelial cells during the proliferative and remodeling phases of wound healing. Upon exposure to a diabetic wound site with bacterial infection, the hydrogel encounters an environment rich in reactive oxygen species and characterized by low pH, indicative of inflammation. This specific environment triggers a rapid dissolution of the boronic ester bonds within the hydrogel structure, causing it to collapse and enabling the staged release of PDA@Ag NPs and rhCol III.

**FIGURE 1:**
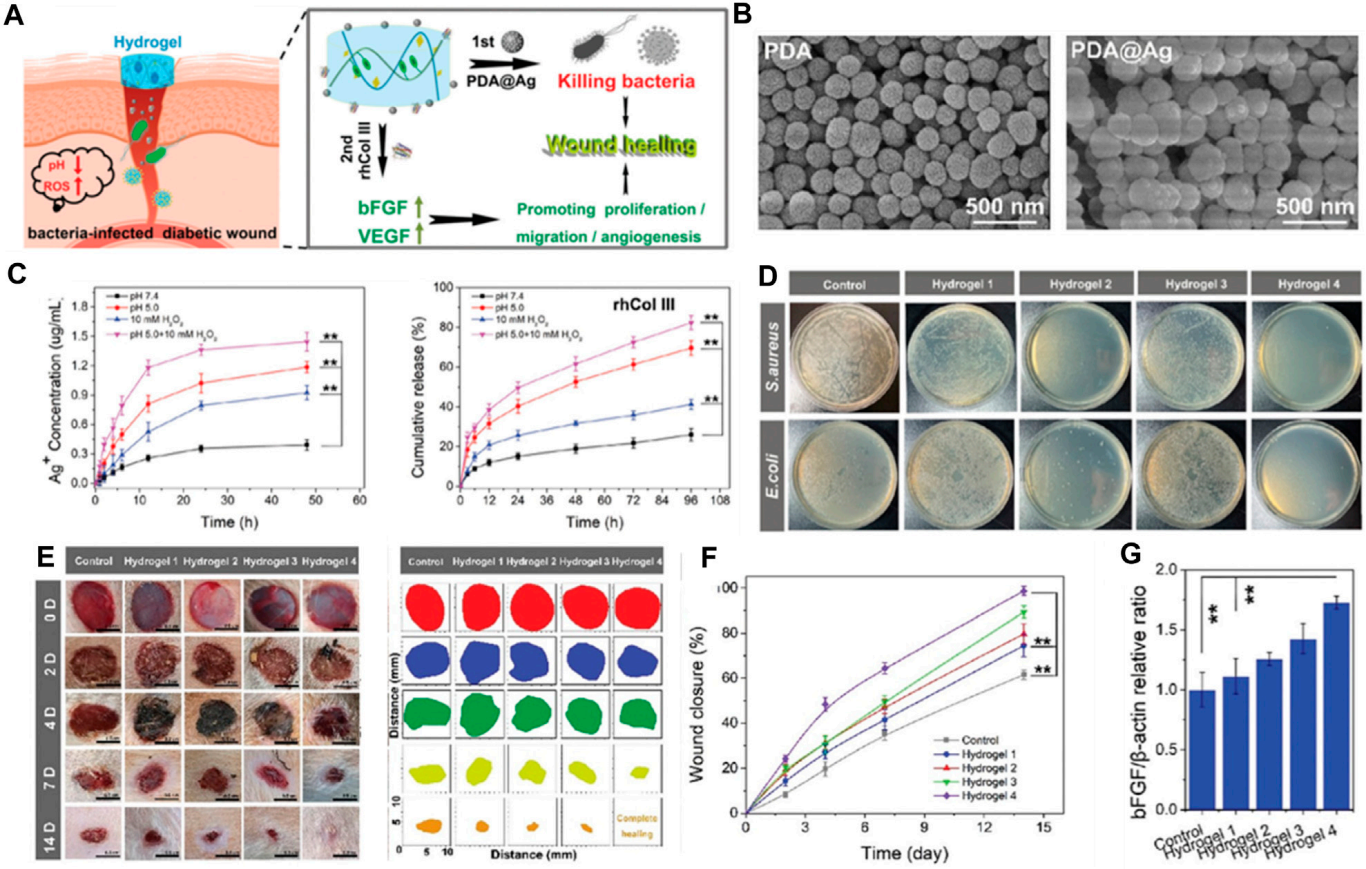
**(A)** The microenvironment-responsive hydrogel and its therapeutic mechanism contributing to the promotion of chronic wound healing. **(B)** The SEM images of for PDA and PDA@Ag NPs. **(C)** The release profiles of payloads from hydrogel. **(D)** Representative images of *S. aureus* and *E. coli* following a 12-h treatment with various hydrogel formulations. **(E)** Illustrative examples of the progression of wound closure on days 0, 2, 4, 7, and 14 (right) after being subjected to different treatments at predetermined time points (left, *n* = 8). **(F)** Over the course of 14 days, the rate of wound contraction was also tracked and quantified. **(G)** The proportional protein expression ratio of bFGF to b-actin. Note the following hydrogel types: (1) Hydrogel 1: Control hydrogel. (2) Hydrogel 2: Hydrogel encapsulating PDA@Ag nanoparticles. (3) Hydrogel 3: Hydrogel encapsulating rhCol III. (4) Hydrogel 4: Hydrogel encapsulating both PDA@Ag and rhCol III. Reproduced with permission from ref Hu et al. (2021).

As a result, the hydrogel framework collapses, facilitating the staged discharge of PDA@Ag NPs and rhCol III (Figure 1A). The Scanning Electron Microscopy (SEM) findings depicted in Figure 1B illustrate the spherical form of both PDA and PDA@Ag NPs. The payloads encapsulated within the hydrogel demonstrate a pH-sensitive release dynamic, where the rate of release notably escalates under the more acidic conditions of pH 5 (Figure 1C). The agar plate counting experiment demonstrated that the hydrogel@Ag&rhCol III group exhibited the most substantial antibacterial efficiency, as indicated by the fewest bacterial colonies (Figure 1D). The efficacy of the hydrogel in facilitating chronic wound healing was evaluated using a rat wound model infected with *E. coli*. Among all groups, the hydrogel@Ag&rhCol III group exhibited the most rapid wound healing, achieving a 64% wound healing rate by day 7 (Figures 1E, F). As presented in Figure 1G, the hydrogel@Ag&rhCol III group exhibited notably elevated levels of bFGF expression compared to other groups. This observation implies that the hydrogel@Ag&rhCol III has the potential to amplify the expression of bFGF, thereby fostering enhanced cell proliferation and angiogenesis.

To summarize, the hydrogel responsive to microenvironmental changes has shown exceptional capabilities in combating bacteria and promoting cell growth and movement, successfully speeding up the healing process of chronic diabetic wounds in both laboratory and real-world scenarios. This research confirms the significant potential of newly designed rhCol III for use in mending and regenerating long-term wounds. As the authers look ahead, they foresee the creation and implementation of further bespoke products based on recombinant human collagen, contributing to advancements in human health and wellbeing.

**FIGURE 2:**
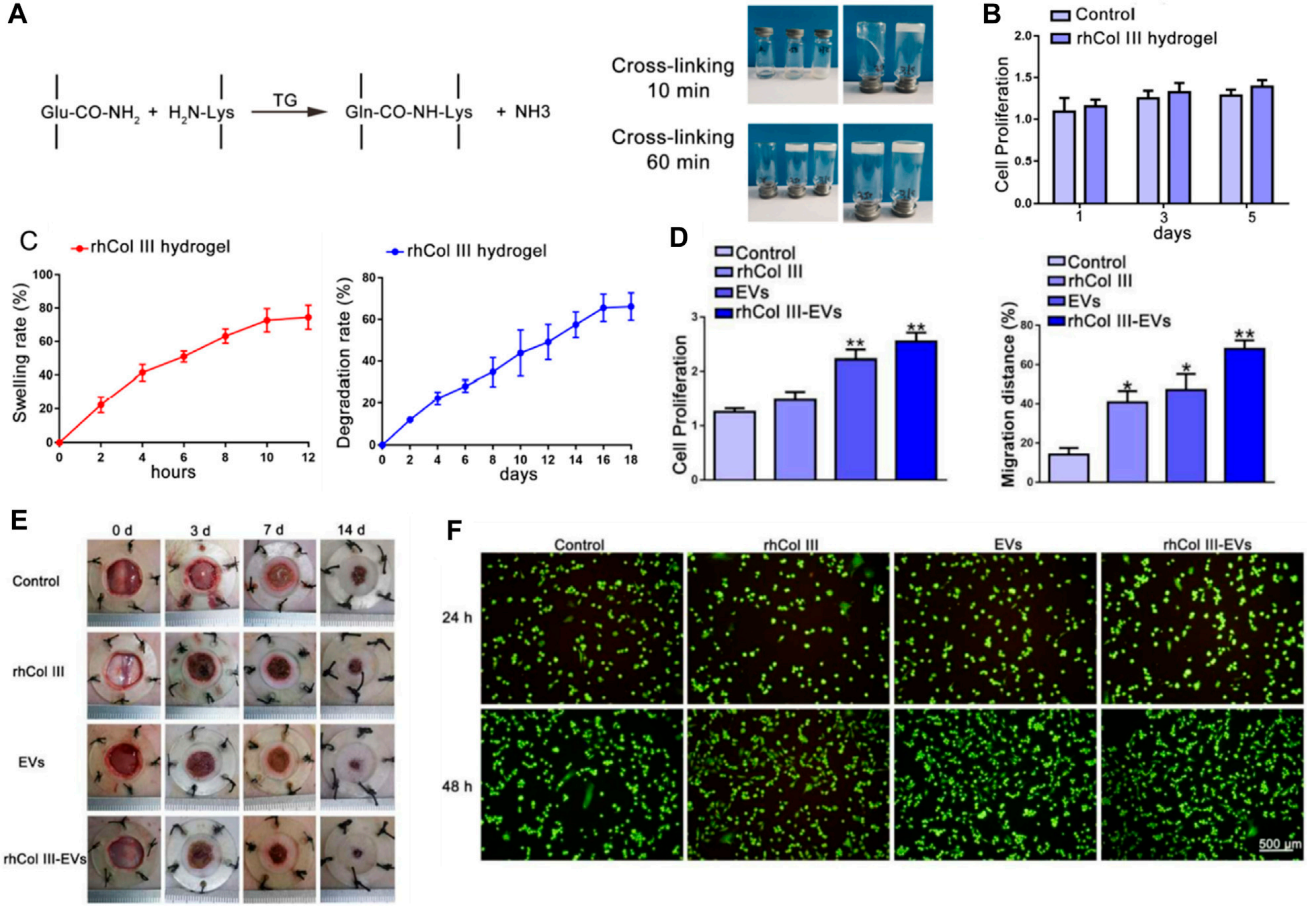
**(A)** Illustrative representation of the procedure for preparing the recombinant human collagen III (rhCol III) hydrogel. **(B)** Impact of the rhCol III hydrogel extract on cellular proliferation. **(C)** Swelling ratio observed in the rhCol III hydrogel and the rate of degradation identified in the rhCol III hydrogel. **(D)** Proliferations and migration distance of L292 cells in rhCol III hydrogel. **(E)** images of wounds from each group, taken at baseline (0 days) and subsequent timepoints (3, 7, and 14 days). **(F)** Comparison of angiogenesis in human umbilical vein endothelial cells (HUVECs) in various groups. Reproduced with permission from ref Xu et al. (2022).

Wang et al. centers on a pivotal study that delves into the application of recombinant human collagen III protein hydrogels and extracellular vesicles (EVs) in skin wound healing (Figure 2A) (Xu et al., 2022). The research team conducted a series of experiments to assess the efficacy of these hydrogels and EVs in promoting wound healing. The findings suggest that the hydrogels capably released the EVs, thereby stimulating cell proliferation, migration, and angiogenesis (Figures 2B–D). Initially, the study underscores the crucial role of skin as a protective barrier for the human body and tackles the challenges associated with the wound healing process, such as diabetes, vascular insufficiency, and local pressure alterations. The authors elucidate that collagen III, a vital component of the extracellular matrix, plays a significant role in wound healing (Figures 2E, F). They delineate the preparation process of recombinant human collagen III protein hydrogels and discuss the role of EVs in sustained therapeutic agent release for wound healing promotion.

The researchers conducted several experiments to determine the efficacy of these hydrogels and EVs in wound healing. The hydrogels successfully released the EVs, enhancing cell proliferation, migration, and angiogenesis. They also suppressed the inflammatory response and promoted wound healing in a diabetic rat skin injury model. The study concludes that these hydrogels and EVs hold significant potential in skin wound healing, presenting a novel approach for chronic wound treatment. In summary, the authors offer an intricate discourse on the employment of hydrogels as a delivery system for recombinant collagen, and the utilization of EVs for sustained therapeutic agent release. They effectively illustrate that the hydrogels proficiently discharge the EVs, thereby facilitating wound healing in a diabetic rat skin injury model. This insight contributes a novel and promising stratagem to the therapeutic repertoire for chronic wound management.

Presently, hydrogels stand as an encouraging scaffold material for tissue engineering and regenerative strategies, largely due to their high-water content, tissue-like mechanical properties, and adjustable physical features (Kathawala et al., 2019; Su et al., 2021). Collagen-based hydrogels, in particular, are appealing for their ability to mimic the extracellular matrix of connective tissues. Recombinant collagen boasts several advantages over tissue-extracted collagen, such as enhanced standardization and tunability, and it circumvents issues of immunogenicity or pathogen transmission (Muhonen et al., 2017). Nevertheless, striking the right balance between factors like swelling, degradation, pore size, and mechanics remains a challenge in optimizing hydrogel design and collagen incorporation. Moreover, the production of most recombinant collagen relies on mammalian cell culture systems, adding a considerable cost (Chen et al., 2020). There is a call for further research to boost recombinant collagen yields and devise efficient purification strategies to curtail expenses (Chen et al., 2022). In summary, while recombinant collagen-loaded hydrogels offer a promising path in tissue repair, further optimization and cost-cutting measures are essential to usher these technologies from the laboratory to clinical practice. Future research should focus on scalable recombinant collagen production, the incorporation of cell instructive signals, and *in vivo* assessment of performance and host response.

## 3 The broad application potential of recombinant collagen in corneal stroma regeneration

Recombinant collagen has emerged as a promising biomaterial for various regenerative medicine applications owing to its versatility, biocompatibility, and improved safety compared to animal-derived collagens (Strauss and Chmielewski, 2017). As the most abundant protein in the human body and a major component of connective tissue, collagen plays a critical role in supporting cell growth, adhesion, and organization during tissue regeneration (Sheehy et al., 2018). Recombinant collagen can be biosynthesized using genetic engineering approaches, allowing precise control over collagen type, structure, degradation kinetics, and functionalization with biological signals (Felician et al., 2018). This advanced engineering of molecular and material properties makes recombinant collagen highly adaptable for developing scaffolds, hydrogel, coatings, and delivery systems tailored to promote regeneration across diverse tissues including skin, bone, cartilage, vasculature, and others (Addi et al., 2017; Quinlan et al., 2017; Yang et al., 2021a). The modular and customizable nature of recombinant collagen, along with its inherent bioactivity and biodegradability, enables the design of therapeutic platforms that synergize with endogenous regenerative processes (McPhail et al., 2020). Further research and clinical translation of recombinant collagen-based therapies holds promise for enabling more effective and safer regenerative medicine solutions. In 2021, Sun et al. developed recombinant human collagen hydrogels with hierarchically ordered microstructures to regenerate corneal stroma (Kong et al., 2022). The RHC are modified with methacrylate anhydride (MA) to mimic native corneal properties. The collagen hydrogels have aligned microgrooves and inverse opal nanopores (MI-RHCMA). *In vitro* experiments show MI-RHCMA hydrogels guided organized growth and differentiation of limbal stromal stem cells into keratocytes compared to random collagen gels. *In vivo* rat studies demonstrated MI-RHCMA implants integrate with host tissue and regenerate damaged corneal stroma better than controls.

**FIGURE 3:**
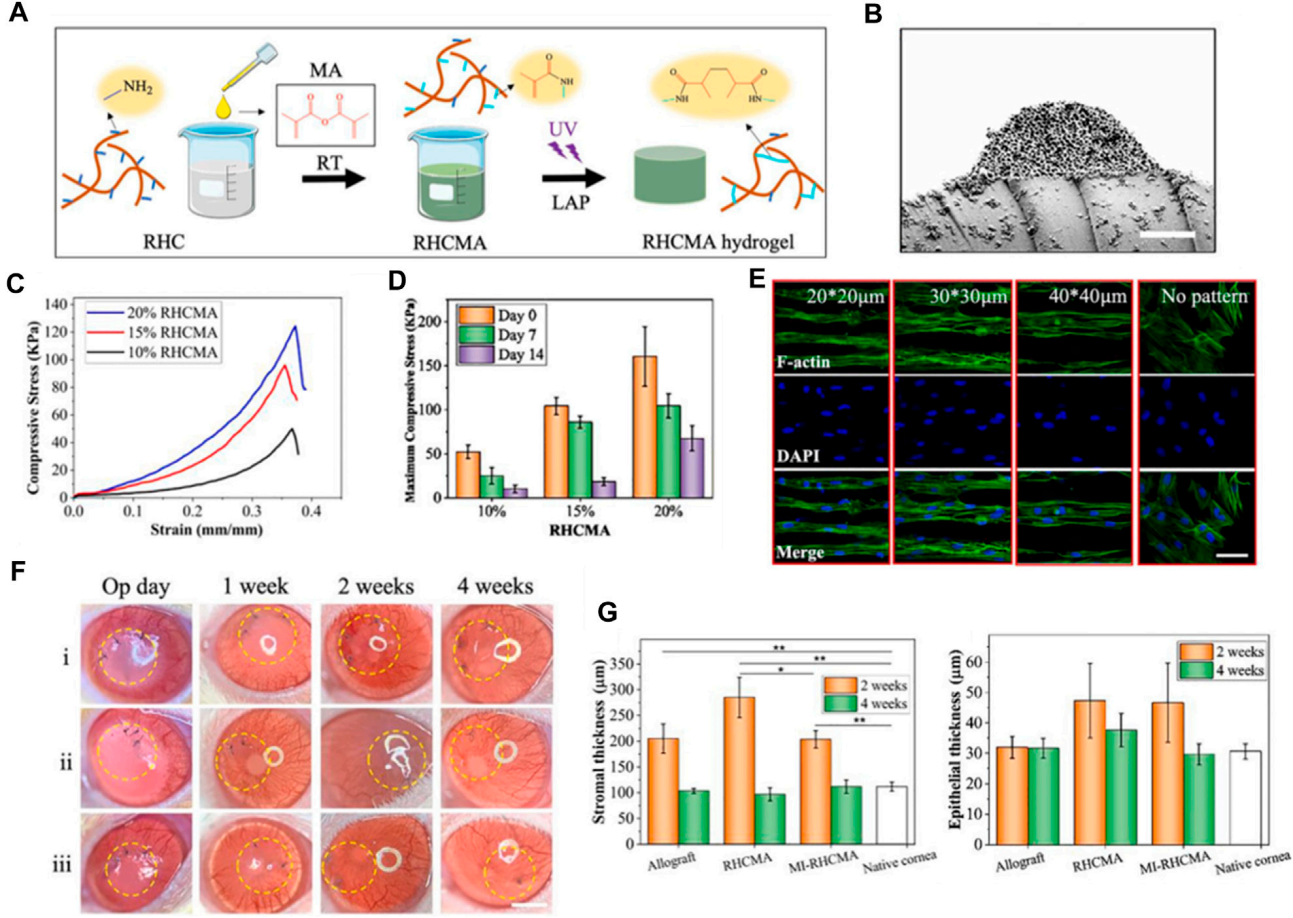
**(A)** Graphic representation of rhcma hydrogel synthesis process. **(B)** Cross-sectional SEM visuals of the mi-RHCMA hydrogel patch. Scale bar, 5 μm. **(C)** Compressive strain-stress relationships of various RHCMA hydrogels post 60-min PBS soak. **(D)** Histograms displaying the peak compressive stress for different RHCMA hydrogels following immersed in PBS. **(E)** Diagram exhibiting the cytoskeletal and nuclear staining in LSSCs hosted on constructs. Scale bar, 5 μm. **(F)** Representative photographs illustrating the post-surgical ocular conditions of corneas that treated with i) allograft, ii) RHCMA, and iii) MI-RHCMA hydrogel patches, captured immediately. Scale bar, 1.5 mm. **(G)** Measurements of the thickness of the stroma **(B)** and the epithelium **(C)** were taken at day 14 and day 28 post-operation for corneas that had undergone transplantation with allograft, RHCMA, and MI-RHCMA hydrogel patches. Reproduced with permission from ref Kong et al. (2022).

In their research, RHCMA was engineered by integrating MA onto the collagen macromolecular chain via a condensation reaction between amino and carboxyl groups, as depicted in Figure 3A. This method effectively maintained the inherent superior biocompatibility of collagen hydrogel. The distinctive structure of the MI-RHCMA hydrogel patch is clearly illustrated in Figure 3B. The cross-sectional view showcases the convexity and indentation of the microgrooves, which further reveal the existence of inverse opal pores within the microgroove. Figures 3C, D provide a graphical representation of the compressive strain-stress relationship, along with the maximum compressive stress experienced by the RHCMA hydrogel when submerged in PBS. When it comes to cell behavior, LSSCs displayed a tendency to form an organized and elongated structure on MI-RHCMA hydrogel patches, contrasting with their random distribution on unpatterned RHCMA hydrogel surfaces, as shown in Figure 3E. The surgical and post-surgical observations are depicted in Figure 3F. MI-RHCMA hydrogel patches were grafted onto the left eyes of rats, with the right eyes serving as controls. These assessments were performed immediately post-surgery and at 1, 2, and 4 weeks following the operation. Figure 3G presents the results of a histological analysis for measuring corneal stromal and epithelial thickness. Interestingly, no significant statistical variation was observed in the thickness of the corneal epithelium across the allograft, MI-RHCMA, RHCMA, and native corneas.

To summarize, researchers fabricated a novel recombinant human collagen hydrogel designed for corneal tissue restoration. The final product was a hierarchically structured hydrogel, crafted through the amalgamation of RHCMA hydrogel, lithography, and photonic crystal techniques. This material, featuring inverse opal nanopores and aligned microgrooves, alongside ordered topological indications, promoted the aligned growth and differentiation of LSSCs into keratocytes *in vitro*. Moreover, RHCMA hydrogels with these organized microstructures were found to boost tissue repair processes and foster the regeneration of damaged stromal tissue *in vivo*. These attributes underscore their promising potential in the domains of tissue repair and stroma regeneration.

Recombinant human collagen (RHC) polypeptide holds a significant edge over natural collagen sources in the realm of tissue engineering applications (Yang et al., 2021b). This is particularly advantageous when compared to animal-derived collagens, as it considerably reduces the risk of immune rejection upon implantation (Parmar et al., 2017). One of the key advantages of RHC is its capacity for precise and customizable biosynthesis. This allows for the engineering of specific peptide sequences, integrin binding sites, growth factors, and cross-linking into the polypeptide chain (Haagdorens et al., 2019; Wang, 2021). Such level of control paves the way for tuning the properties of RHC to achieve optimal performance in specific applications. In addition, the production of RHC yields a highly consistent and reproducible biomaterial, thereby ensuring uniformity in its quality (Wang, 2021). This process also eradicates risks associated with pathogen transmission from animal sources and negates the need for reliance on animal harvesting, thus providing an abundant, sustainable supply of human collagen (Rico-Llanos et al., 2021). The degradation rate of RHC is tunable, and it boasts processing versatility, and overall customizability, which further enhances its suitability as a biomaterial. These properties make RHC an ideal biomaterial for the development of engineered tissues and scaffolds for various applications. These include, but are not limited to, skin grafts, tendon/ligament repair, wound healing, and other regenerative medicine applications.

The potential for employing scaffolds as vehicles for recombinant collagen in the fields of tissue engineering and regenerative medicine is considerable. These scaffolds deliver a three-dimensional construct reminiscent of the natural extracellular matrix, fostering cellular attachment, proliferation, and differentiation. Scaffolds comprising recombinant collagen present numerous benefits compared to conventional scaffolds manufactured from animal-derived collagen. The former can be produced on a large scale, with meticulous regulation of composition and purity (He et al., 2018). Additionally, recombinant collagen scaffolds can be functionalized with elements such as cell-binding motifs, growth factors, and other biologically active molecules to enhance their efficacy.

Nevertheless, significant obstacles remain. Emulating the intricate architecture and diverse protein composition of native ECM continues to be a challenging task. Matching the degradation rate of scaffolds with the pace of cell/tissue growth persistently proves difficult (Jeon et al., 2017). Engineering tissues over 1 mm in thickness necessitates innovative strategies for vascularization. Moreover, understanding how the physicochemical properties of scaffolds impact cell behavior is still lacking. Current research pursuits are focused on deepening our understanding of cell-matrix interactions, designing innovative biomaterials and processing methodologies, and augmenting the functional characteristics of engineered tissues (Huang et al., 2018). In conclusion, while recombinant collagen scaffolds represent a promising avenue for regenerative medicine, additional research is required to enhance scaffold bioactivity, degradation, and integration within host tissues.

## 4 Utilization of recombinant human collagen in bone tissue repair

Recombinant human collagen has emerged as a significant asset in the field of bone regenerative engineering (Andrews et al., 2019; Fushimi et al., 2020). RhCOL retains the biological attributes of natural collagen while circumventing the issues associated with immunogenicity and pathogen transmission. RhCOL scaffolds facilitate the adhesion, proliferation, and differentiation of osteoblasts *in vitro*. *In vivo* studies illustrate enhanced bone regeneration when rhCOL is used in conjunction with bone marrow-derived mesenchymal stem cells (BMSCs) and/or osteogenic growth factors (Chan et al., 2017).

The composition and structure of rhCOL scaffolds can be precisely tailored to mimic the native bone extracellular matrix. This is achieved through manipulation of collagen crosslinking, mineral content, and the incorporation of bioactive motifs, enabling a controlled degradation rate that synchronizes with new bone deposition (Bien et al., 2020). Moreover, rhCOL scaffolds surpass the limitations of traditional bone graft materials by supporting cellular growth and providing precise control over structural and functional properties (Muhonen et al., 2017; Bien et al., 2020). Ongoing research endeavors aim to optimize integration and healing outcomes as rhCOL transitions from laboratory research to clinical implementation for bone engineering applications.

Sandri et al. delves into the creation of a synthetic bone substitute that emulates the biochemical and biophysical cues intrinsic to the native bone extracellular matrix (Ramírez-Rodríguez et al., 2017). The investigative team employed a recombinant collagen-based scaffold, which was enriched with the tri-amino acid sequence arginine-glycine-aspartate (RGD), aiming to bolster the interaction and differentiation of mesenchymal stem cells. The study witnessed promising progress in the generation of superior quality bone grafts, a feat achieved through biomimetic mineralization of synthetic engineering peptides under the influence of magnesium ions. The novelty of this study hinges on the application of a synthetic bone substitute that, across all scales from macro to nano, replicates the biochemical and biophysical cues of the bone extracellular matrix.

**FIGURE 4:**
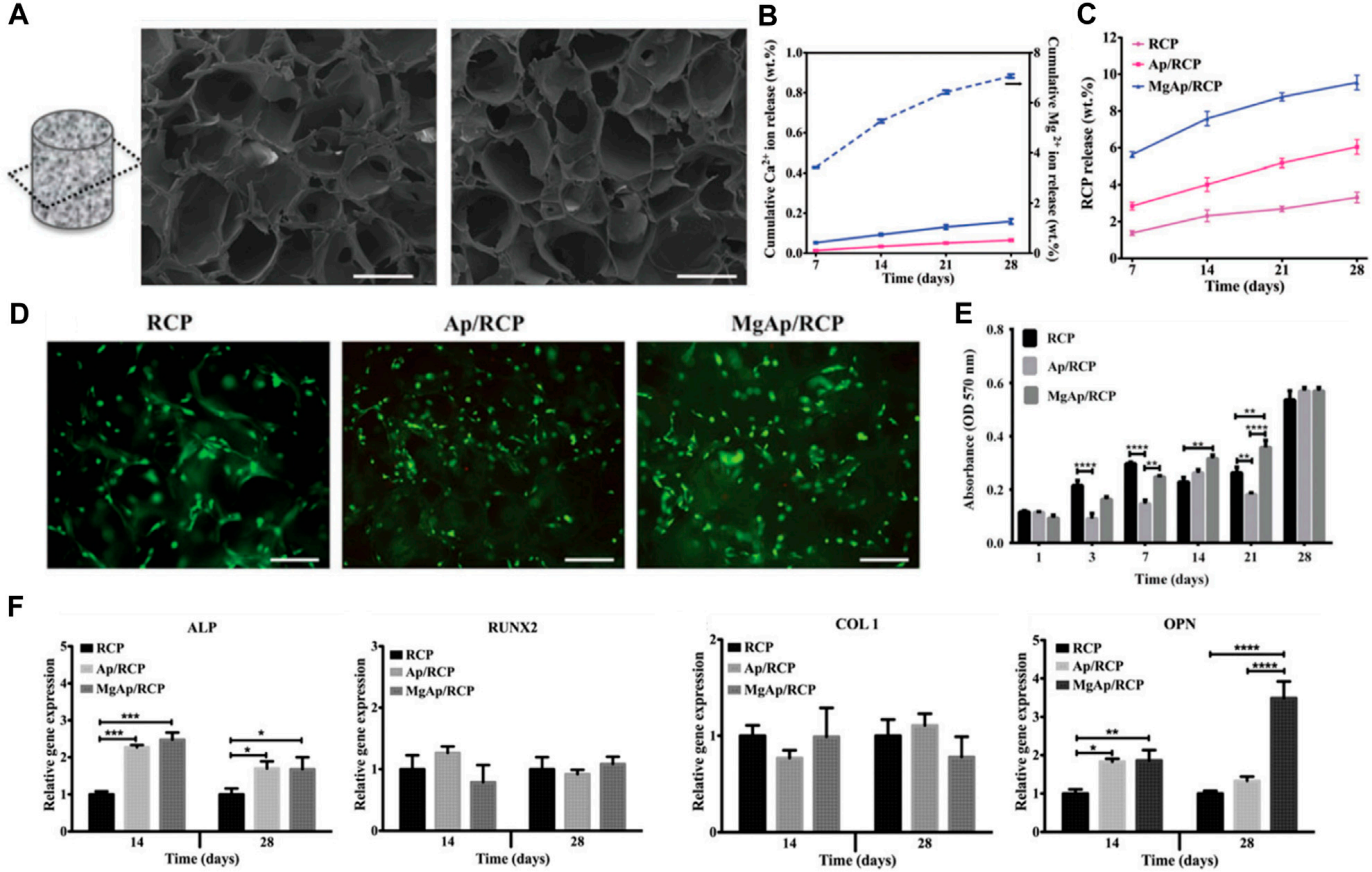
**(A)** SEM analysis of MgAp/RCP scaffolds. Scale bar, 250 mm. Release profiles of **(B)** Ca^2+^ and **(C)** RCP from MgAp/RCP scaffolds. **(D)** Staining of live and dead cells was performed for the investigation of cytocompatibility. **(E)** The MTT assay was employed to evaluate cell viability at multiple time points: after 1, 3, 7, 14, 21, and 28 days. **(F)** mRNA expression investigation of alkaline phosphatase (ALP), collagen I (COL1), osteopontin (OPN) and runtrelated transcription factor 2 (RUNX2). Reproduced with permission from ref Ramírez-Rodríguez et al. (2017).

Three distinct scaffold compositions were characterized by SEM analysis, non-mineralized (RCP), mineralized (Ap/RCP), and mineralized alongside magnesium (MgAp/RCP) possess a highly porous structure with interconnected pores. The mineralized scaffolds exhibit a more compact structure compared to their non-mineralized counterpart, and the incorporation of magnesium leads to a more uniform and homogeneous structure (Figure 4A). Encapsulated Ca^2+^ and Rcp display a consistent and prolonged release pattern (Figures 4B, C). The scaffold demonstrates superior cytocompatibility, exhibiting no adverse or toxic effects on cells (Figure 4D). Detailed examination revealed that the MgAp/RCP scaffolds exhibited the most pronounced MSC proliferation within 28 days (Figure 4E). Through the use of qPCR, they analyzed the messenger RNA (mRNA) levels of ALP, RUNX2, OPN, and COL1, aiming to discern the influence that the three types of scaffolds exerted on the expression of osteogenic markers. Scaffolds exert a significant influence on the expression levels of these mRNAs (Figure 4F).

In 2018, Farrell et al. presented a research investigation focused on developing a novel *in situ* gelling hydrogel, embedded with recombinant collagen peptide microspheres (Fahmy‐Garcia et al., 2018). This unique slow-release system is designed to stimulate ectopic bone formation. The study’s objective was to introduce a promising solution for extensive bone defect repair, employing natural biomaterials which are biodegradable, biocompatible, and can actively interact with the extracellular matrix and cells. The injectable formulation simplifies application and can potentially expedite patient recovery time.

**FIGURE 5:**
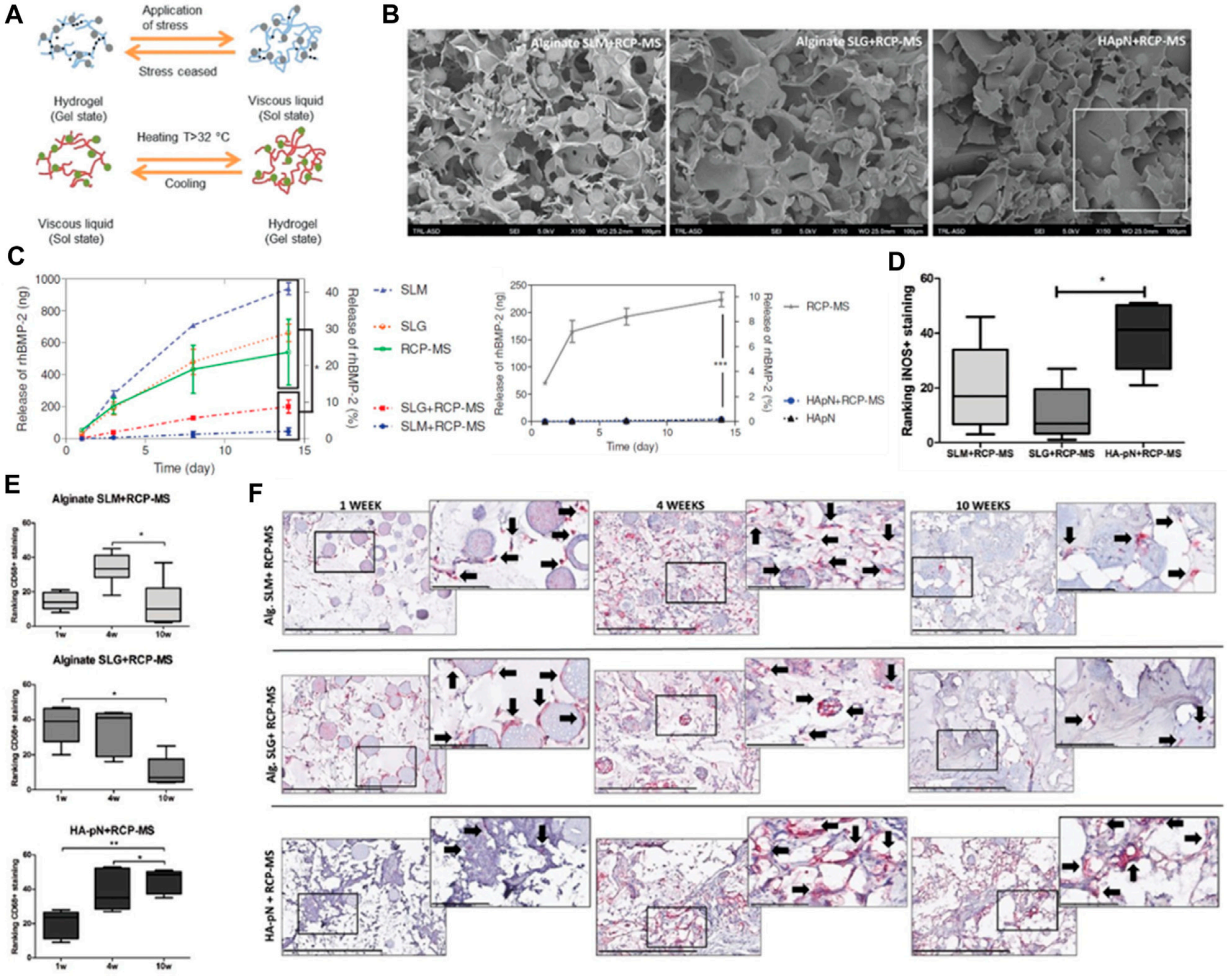
**(A)** The diagram depicts the structural network of a hydrogel infused with microspheres. **(B)** SEM visuals of the SLM+RCP-MS, SLG+RCP-MS, and HApN+RCP-MS formulations are presented. Scale bar, 100 μm. **(C)** The dispersion of BMP-2 is contrasted among various precursors. **(D)** Positive iNOS staining observed in the formulations after a period of 10 weeks. **(E)** Two impartial reviewers classified the implants based on the occurrence of CD68 positive staining observed in the formulations at the intervals of 1, 4, and 10 weeks. **(F)** Representative sample from each implant formulation at 1 and 10 weeks, with a scale bar indicating 400 μm. Reproduced with permission from ref Fahmy‐Garcia et al. (2018).

The research process comprised the production of the hydrogel and microspheres, succeeded by *in vitro* and *in vivo* examinations to assess their properties and effectiveness. The hydrogel was synthesized using a blend of gelatin, hyaluronic acid, and β-glycerophosphate (Figure 5A), while the microspheres were fashioned using recombinant collagen peptide and poly (lactic-co-glycolic acid) (PLGA). Various techniques, including scanning electron microscopy (Figure 5B), Fourier-transform infrared spectroscopy, and rheological analysis, were employed to characterize the hydrogel and microspheres. Alginate hydrogels containing RCP-MS demonstrated a slower release rate, indicating the synergistic effect of microspheres and hydrogels in controlling the release. During the *in vitro* experiments, the hydrogel and microspheres’ biocompatibility and osteogenic potential were evaluated. The findings indicated that the hydrogel and microspheres promoted cells proliferation and differentiation into osteoblasts, suggesting their potential for bone tissue engineering applications. *In vivo* experiments evaluated the hydrogel and microspheres’ efficacy in stimulating ectopic bone formation in a rat model. The results revealed significant enhancement in bone formation in comparison to the control group, as substantiated by micro-computed tomography and histological analysis. The hydrogel and microspheres also facilitated the infiltration of immune cells, including macrophages and M2-like macrophages, which play an essential role in bone regeneration.

In summary, the research demonstrated the potential of the novel *in situ* gelling hydrogel loaded with recombinant collagen peptide microspheres as a slow-release system to induce ectopic bone formation. Both the hydrogel and microspheres displayed excellent biocompatibility and osteogenic potential *in vitro* and significantly augmented bone formation *in vivo*. The findings indicate that this injectable formulation could serve as a promising solution for extensive bone defect repair by leveraging natural, biodegradable, and biocompatible biomaterials that interact with the extracellular matrix and cells.

## 5 Application of recombinant human collagen in 3D bioprinting

Recombinant human collagen has attracted growing interest in 3D bioprinting due to its biocompatibility, low immunogenicity, and customizable biochemical and mechanical properties (Lee et al., 2019; Osidak et al., 2020; Muthusamy et al., 2021). Studies have engineered recombinant human collagen with tailored supramolecular assemblies, crosslinking densities, and matrix stiffnesses to resemble native extracellular matrices (Włodarczyk-Biegun and Del Campo, 2017; Hong et al., 2018). This permits precise control over microenvironments for directing cell fate processes (Tytgat et al., 2020). Moreover, recombinant collagen allows incorporation of cell-adhesive peptides, growth factors, and cytokines to modulate cell behaviors. Currently, recombinant human collagen-based bioinks have been utilized to bioprint tissue constructs such as skin, cartilage, bone, blood vessels, and liver (Zhang et al., 2021). Looking ahead, recombinant human collagen bioinks hold great promise for fabricating complex heterogeneous tissues with biomimetic architectures, compositions, and functions (Gungor-Ozkerim et al., 2018; Isaacson et al., 2018; Gudapati et al., 2020). However, challenges remain in scalable recombinant collagen production and developing universal crosslinking strategies to enhance print fidelity (Martyniak et al., 2022). Further interdisciplinary research on optimizing recombinant human collagen designs, crosslinking mechanisms, and printing processes is critical to enable wide clinical translations of 3D bioprinted tissues and organs (Stepanovska et al., 2021).

**FIGURE 6:**
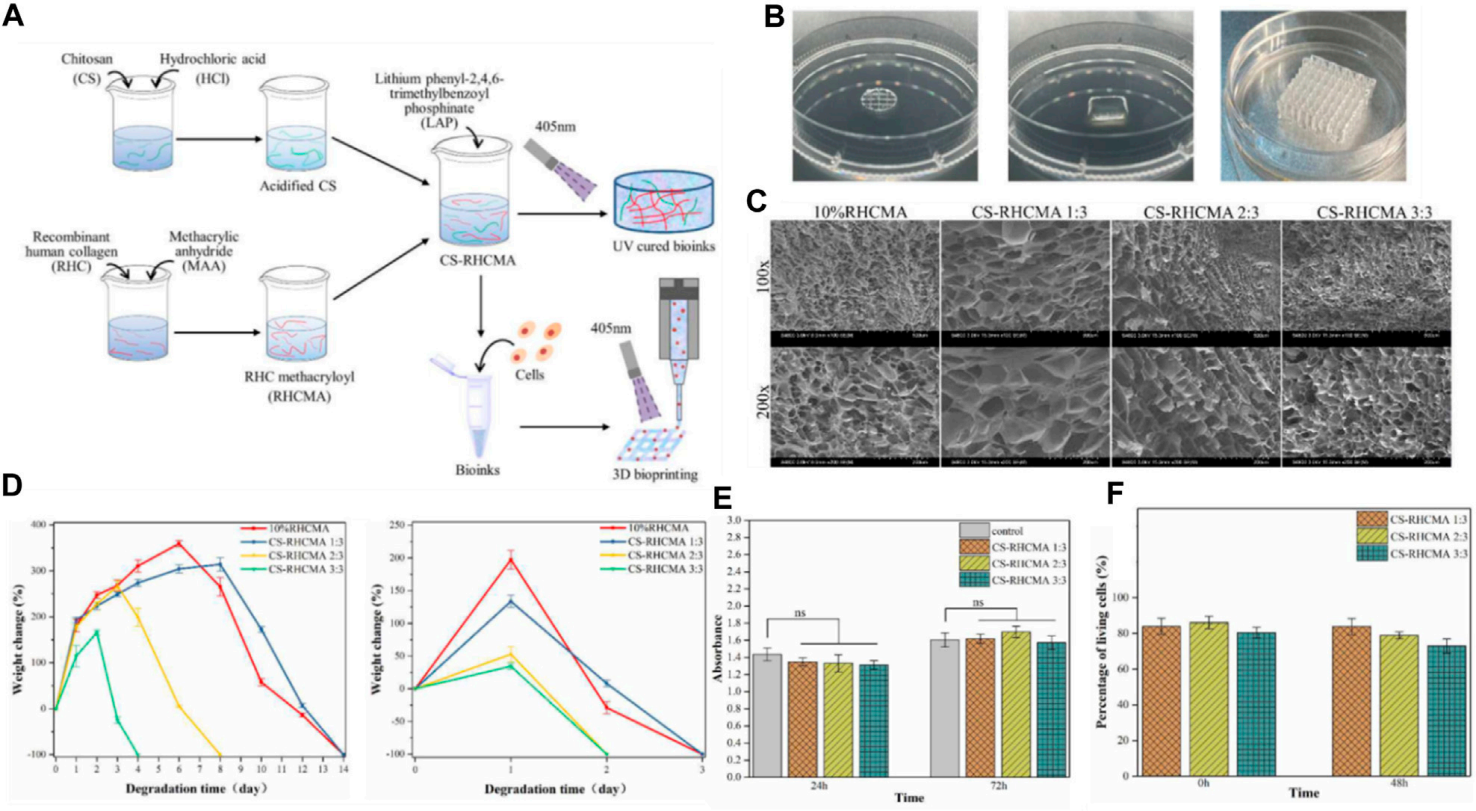
**(A)** Diagrammatic representation of the CS-RHCMA bioinks preparation process. **(B)** Different structures created by 3D printed constructs. **(C)** SEM images of the internal morphology of freeze-dried RHCMA and CS-RHCMA samples. Scale bar, 200 µm. **(D)** Degradation of RHCMA and CS-RHCMA samples when incubated in PBS solution (pH 7.2) and lysozyme solution (pH 6.5), respectively. **(E)** Assessment of HUVECs viability cultured in sample extracts at timeframes of 24 and 72 h. None significant (ns) indicates *p* > 0.05. **(F)** Evaluation of *in vitro* biocompatibility for HUVECs-laden CS-RHCMA bioinks post-printing. Reproduced with permission from ref Yang et al. (2022a).

In 2022, Jin et al. presents the formulation of photo-responsive bioinks based on chitosan and recombinant human collagen for 3D bioprinting (Yang et al., 2022a). The authors delve into the merits of employing these materials, including their biocompatibility, biodegradability, and their proficiency to foster cell proliferation and differentiation. They underscore the cruciality of managing shear stress during the printing operation to preserve the integrity of stem cells (Figure 6).

Type-III recombinant human collagen methacryloyl/acidified chitosan (CS-RHCMA) bioinks were synthesized by incorporating acidified chitosan into a RHCMA solution. The RHCMA was derived by altering recombinant human collagen with methacrylic anhydride. The CS-RHCMA composites were created by amalgamating the acidified chitosan with the RHCMA solution in varying proportions, facilitating the adjustment of the bioinks’ mechanical resilience and internal pore dimensions. The integration of chitosan into RHCMA enhanced the printability of the bioinks, yielding well-structured 3D constructs via extrusion-based 3D printing (Figure 6A). Figure 6B shows different structures created by 3D printed constructs using the CS-RHCMA bioinks. The authors demonstrate the versatility of the bioinks by creating various structures, including a honeycomb structure, a spiral structure, and a grid structure. The printed HUVECs are well sustained within the lattices prepared from the CS-RHCMA samples, with nearly 80% of the cells being alive after the extrusion-based printing. This suggests that the CS-RHCMA bioinks are suitable for 3D bioprinting and can support the growth and viability of HUVECs. SEM images present the internal structure of freeze-dried RHCMA and CS-RHCMA samples. Both samples show a uniformly distributed, interconnected pore structure (Figure 6C). Introducing chitosan to RHCMA increased pore size: average pore size in CS-RHCMA 1:3 is 128 μm, larger than 66 μm in 10% RHCMA. Further CS content increases lead to smaller pores and denser pore walls, with CS-RHCMA 3:3 having the smallest size of 58 μm. These findings indicate that adding chitosan to RHCMA allows control over the bioinks’ internal structure, influencing the mechanical properties and cell behavior in printed constructs. Figure 6D depict the degradation of RHCMA and CS-RHCMA in PBS (pH 7.2) and lysozyme solution (pH 6.5), measured by weight loss over time. Lysozyme-incubated samples degraded completely in 4 days, while those in PBS took 14 days. RHC and CS-RHCMA 1:3 degraded slower but increasing CS ratio sped up degradation. This is likely due to changes in mechanical properties from added acidified CS disrupting gelation networks. Thus, higher strength UV-cured bioinks resist fast breakdown, and degradation rate can be adjusted by varying bioink composition, influencing the stability and longevity of printed constructs. Figure 6E demonstrates that all the UV-cured CS-RHCMA bioinks were cytocompatible and suitable for 3D bioprinting *in vitro*.

The findings affirm the cytocompatibility of UV-cured CS-RHCMA bioinks, rendering them suitable for *in vitro* 3D bioprinting. The viability of cells within the bioprinted lattice approximated 80%, underscoring the bioinks’ ability to foster HUVECs growth and survival (Figure 6F). These insights are pivotal in advancing bioinks for 3D bioprinted vascularized tissues and organ constructs.

The novelty of this research rests on the generation of a photo-responsive bioink capable of fabricating intricate 3D constructs with superior resolution and cell viability. The authors illustrate the promise of this bioink for tissue engineering applications, encompassing the production of skin and cartilage tissues.

Nonetheless, this work is not without its challenges. These include the necessity for further refinement of the printing protocol and the imperative to scale up manufacturing for clinical use. Moreover, the authors acknowledge that additional research is requisite to fully comprehend the enduring impacts of employing these materials in a living organism.

Recombinant human collagen has garnered interest in 3D bioprinting due to its biocompatibility, low immunogenicity, and adaptable biochemical and mechanical traits (Elalouf, 2021). Research has tailored RHC to mimic native extracellular matrices with custom supramolecular assemblies, crosslinking densities, and matrix firmness, allowing precise control over cellular microenvironments for directing cell fate (Cui et al., 2017). Furthermore, RHC allows the integration of cell-adhesive peptides, growth factors, and cytokines to influence cell behaviors (Dai et al., 2021). At present, RHC-based bioinks are used to bioprint various tissue constructs, including skin, cartilage, bone, blood vessels, and liver (Tang et al., 2021). Moving forward, RHC bioinks possess significant potential for crafting complex, heterogeneous tissues with biomimetic structures, compositions, and functionalities (Zhang et al., 2017; Matai et al., 2020). Nevertheless, hurdles persist in scalable RHC production and devising universal crosslinking strategies for improved print fidelity. Continued interdisciplinary research on refining RHC designs, crosslinking mechanisms, and printing methodologies is crucial for broad clinical translation of 3D bioprinted tissues and organs.

## 6 Conclusion and discussion

The production of recombinant human collagen (RHC) is a complex biotechnological process that encompasses the utilization of specific host cells modified to express human collagen genes. The process begins with the isolation of the relevant human genes encoding collagen, which are then cloned into vectors–DNA molecules capable of carrying foreign DNA into a host cell. These vectors are subsequently introduced into host cells such as *Escherichia coli*, yeast, or mammalian cells, which have been chosen based on their ability to produce collagen in a form that retains its native structure and function (He et al., 2018; Sheehy et al., 2018).

Following transformation, the host cells are cultured in a controlled environment that is optimized for the expression of the collagen gene. The production involves the scaling up of cell cultures in bioreactors, where conditions such as temperature, pH, and nutrient supply are meticulously managed to maximize yield and product quality (Włodarczyk-Biegun and Del Campo, 2017; Lee et al., 2019). Post-translational modifications crucial for collagen stability and function, such as hydroxylation and glycosylation, are carefully orchestrated within the production system.

The benefits of using RHC instead of animal-derived collagen are multifaceted (Radke et al., 2018; Lagali, 2020; Elalouf, 2021): (1) safety: RHC reduces the potential for zoonotic disease transmission and immunogenic reactions as it is produced in a controlled environment without sourcing from animal tissues; (2) Consistency: the production of RHC can be tightly regulated to ensure batch-to-batch consistency, which is a significant challenge with animal-derived collagen due to natural biological variability; (3) Customization: RHC can be modified at the genetic level to include specific amino acid sequences or to introduce particular post-translational modifications, which is not feasible with animal-derived collagen. This allows for the creation of collagen with precise characteristics required for specific applications. (4) Ethical Considerations: RHC production avoids the ethical concerns associated with the use of animal products.

It is important to highlight that customizing animal-derived collagen is inherently challenging. The extraction process from animal tissues can lead to batch variability, and the complexity of the native collagen structure makes it difficult to modify post-translationally. This results in a product that may not be consistently reliable for precise biomedical applications, where uniformity in structure and function is paramount (Osidak et al., 2020).

RHC has emerged as a biomaterial with extraordinary versatility and promise in the realms of tissue engineering and regenerative medicine, presenting a multitude of benefits over traditional animal-derived collagens. Its uniform composition, markedly reduced immunogenicity, and the amenability to molecular engineering for bespoke applications highlight its broad potential in diverse biomedical applications (Nocera et al., 2018). Research has underscored the adaptability of RHC in various formulations such as hydrogels, scaffolds, and lyophilized substances, which have been successfully applied in healing wounds, regenerating skin, and reconstructing osseous and cartilaginous tissues.

The ability to enhance these RHC-based materials with growth factors, cellular elements, or nanoparticles opens up avenues for precision customization, aligning material properties with the nuanced demands of specific therapeutic contexts. The efficacy of RHC is evident in its application to skin grafts, weight-bearing tissue repair, and the engineering of vascular grafts—areas where there is substantial documentation of its success, particularly in preclinical animal studies (Curtin et al., 2018; Elalouf, 2021; Yang et al., 2022a).

To expand upon this, the future of RHC research is poised to delve into next-level innovations that may redefine therapeutic approaches. For instance, the integration of RHC with cutting-edge bio-fabrication technologies, such as 3D bioprinting, has the potential to construct tissues and organs with unprecedented complexity and functionality (Liverani et al., 2017; Rico-Llanos et al., 2021). This would not only revolutionize how we approach complex tissue reconstruction but also hold implications for personalized medicine, where RHC-based tissues are tailored to individual patient’s biological profiles.

Moreover, there exists a burgeoning interest in exploring the synergistic combinations of RHC with synthetic polymers, which may yield composite materials with enhanced mechanical properties and biological functionalities. Such composites could offer new solutions for the regeneration of tissues that require a high degree of biomechanical resilience, such as in the case of intervertebral disc repair or the reconstruction of load-bearing joints (He et al., 2018; Rico-Llanos et al., 2021; Binlateh et al., 2022).

Another prospective area of RHC application lies in the realm of controlled drug delivery systems (Lagali, 2020; Yang et al., 2022b). By embedding therapeutic agents within RHC matrices, it may be possible to achieve localized, sustained release of drugs at injury sites, thereby enhancing the healing process while minimizing systemic side effects.

The ongoing research and future explorations are expected to not only address the current limitations surrounding RHC production and application but also to unlock novel therapeutic paradigms. As we stand on the cusp of these scientific advancements, RHC research is truly at an inflection point, with the anticipation that future studies will bring forth groundbreaking applications that will cement RHC’s status as an invaluable asset in biomedical engineering and beyond. (Liverani et al., 2017).

RHC is an area of intense research interest due to its potential applications in biomedicine, particularly in tissue engineering and regenerative medicine (Parmar et al., 2017; Yang et al., 2021b; Gajbhiye and Wairkar, 2022). The future direction of RHC-related research is shaped by the need for safer, more effective, and customizable biomaterials. Here are several promising avenues for future research:

### 6.1 Enhanced biomimicry

Future research will likely focus on improving the biochemical and biomechanical properties of RHC to more closely mimic the native characteristics of human collagen. This includes fine-tuning the amino acid composition, crosslinking patterns, and molecular alignment to replicate the mechanical strength and biological signaling present in the human body.

### 6.2 Genetic engineering advances

Advancements in genetic engineering techniques can be applied to modify the genes used to produce RHC, leading to collagens with specific properties or functions that are difficult to obtain from natural collagens. This can enable the custom design of collagen molecules for specific medical applications.

### 6.3 3D Bioprinting integration

The integration of RHC with advanced 3D bioprinting techniques is an exciting frontier. Research will likely explore the development of specialized bioinks that can be used to print complex, multicellular tissues and organs with high precision.

### 6.4 Smart biomaterials

The development of “smart” RHC-based materials that can respond to physiological stimuli, such as changes in pH or temperature, could revolutionize drug delivery systems and dynamic tissue scaffolds that adapt to the healing process.

### 6.5 Personalized medicine applications

With the advent of personalized medicine, RHC could be tailored to individual patients based on their genetic makeup, potentially improving the outcomes of treatments and reducing the risk of adverse reactions.

By addressing these future directions, RHC research can contribute to creating more effective therapeutic strategies and innovative solutions for complex medical challenges.

However, challenges persist. To fully realize the potential of RHC, high-yield, cost-effective production systems need to be established (Fu et al., 2019; Groetsch et al., 2019). Systems based on mammalian cells are often associated with low yields and high costs, while bacterial and yeast systems, although more cost-effective, may not achieve the necessary post-translational modifications. Innovations in genetic manipulation and enzyme supplementation may offer potential solutions to these problems (Radke et al., 2018). The use of RHC in 3D bioprinting also invites further exploration. As technologies evolve, RHC’s potential to be used in the fabrication of complex, heterogeneous tissues with biomimetic architectures, compositions, and functionalities could be transformative.

In summary, the research summarized in this review underscores the significant potential of RHC in tissue engineering and regenerative medicine. Despite remaining challenges, the progress made so far in the development and application of RHC is encouraging, and the future of RHC looks promising. As the field continues to advance, RHC is likely to play an increasingly important role in the development of improved biomaterials, therapies, and patient outcomes.
